# Reproductive experience influences the effects of *Lactocaseibacillus rhamnosus* HN001 on gut microbiota and hippocampal plasticity in female rats

**DOI:** 10.1111/jne.70068

**Published:** 2025-07-22

**Authors:** Jodi L. Pawluski, Khadidja Kacimi, Cai Zhang, Laetitia Guillot, Aliocha Lo Guidice, Thierry D. Charlier, Joseph S. Lonstein

**Affiliations:** ^1^ Univ Rennes, INSERM, EHESP, IRSET (Institut de Recherche en Santé, Environnement et Travail), UMR_S 1085 Rennes France; ^2^ Univ Rennes, Uni Caen, CNRS, EthoS (Éthologie Animale et Humaine), UMR 6552 Rennes Cedex France; ^3^ Neuroscience Program & Department of Psychology Michigan State University East Lansing Michigan USA

**Keywords:** hippocampus, matrescence, microbiome, postpartum, probiotic

## Abstract

There is increasing interest in the role of probiotics in supporting maternal well‐being throughout female reproduction. However, it remains largely unknown whether the brain of a female with reproductive experience responds differently to probiotics compared to females without reproductive experience. Reproduction involves remarkable neuroplasticity; therefore, we hypothesized that reproducing females are particularly susceptible to the effects of probiotic treatment. Groups of early pregnant or age‐matched virgin female Long–Evans rats were administered the probiotic, *Lactocaseibacillus rhamnosus* HN001 (HN001), in their drinking water or given untreated water for 30 days. To measure changes in gut microbiota, fecal samples were taken regularly. Brains were analyzed at the end of treatment to quantify hippocampal cells containing the neurogenesis marker doublecortin, the synaptic marker synaptophysin, and the microglial activation marker Iba1. For dams, an offspring retrieval test was performed. Main findings show that HN001 administration lowers *Bacteroidota* abundance in the gut regardless of reproductive experience. In HN001‐treated dams there was an increase in the number of times offspring were carried and this was negatively correlated with *Bacteroidota* abundance in the dam's gut. HN001‐treated dams also had more immature neurons in the hippocampus and more thick‐type microglial cells in the dorsal hippocampus compared to control dams. HN001‐treated females, regardless of reproductive experience, had lower density of synaptophysin immunoreactivity in the CA1, and more thick‐type microglia cells in the ventral hippocampus, compared to control females. These results indicate that the probiotic, HN001, alters female rat maternal behavior, plasticity in the hippocampus, and the gut microbiota abundance, with some effects being influenced by reproductive experience.

## 1 INTRODUCTION

There is growing interest in targeting the maternal gut‐microbiota–brain axis to promote maternal mental health. The gut microbiota are billions of microorganisms that are linked to health and disease by producing metabolites and neurotransmitters that are important factors not only for the gut, but also for the brain.^1^, ^2^ Targeting the gut microbiota to maintain and promote mental health specifically during the peripartum period has been proposed over the past few years.^3^, ^4^, ^5^, ^6^, ^7^, ^8^, ^9^ However, little research has investigated how altering the gut microbiota affects plasticity in the brain of females^6^, ^10^ and whether these effects differ with reproductive experience.

We know that the diversity and abundance of different gut microbes change across pregnancy and the postpartum period.^11^, ^12^ Although there is no consensus on the normative changes in gut microbiota across pregnancy, gut microbes likely play a role in maternal health and illness.^12^ It is also thought that targeting the gut microbiota during pregnancy with probiotics can improve both maternal and fetal outcomes.^7^, ^8^ Probiotics are living microorganisms that, when given in adequate amounts, can have various health benefits.^13^ As probiotics are generally considered safe for use during pregnancy and are readily available,^13^ a growing number of pregnant women are taking probiotic supplements.^14^ Research shows that probiotics may provide health benefits for a number of pregnancy‐related illnesses, including mental illness,^7^, ^8^, ^13^ but because probiotic supplements almost always contain a mixture of microbes, it remains to be determined which microbes contribute to which specific health benefits.

We recently investigated the impact of the probiotic *Lacticaseibacillus rhamnosus* HN001 (HN001) (formerly named *Lactobacillus rhamnosus* HN001) on maternal affective behaviors, offspring‐directed care, and the gut‐microbiota–brain axis in laboratory rat dams.^10^ HN001 is one of the most well‐known probiotic strains and has shown efficacy in promoting gut health and mental health in humans.^15^, ^16^, ^17^ Our research in rats revealed that oral HN001 administration during pregnancy and through the early postpartum period reduced maternal anxiety‐related behavior as well as altered prefrontal cortical levels of serotonin, dopamine, and norepinephrine.^10^ These findings complemented and expanded previous clinical research showing significant reductions in postpartum depressive and anxiety symptoms in human mothers following daily ingestion of HN001 starting in the second trimester of pregnancy and continuing through 6 months postpartum.^18^ Interestingly, another study, where women ingested a probiotic consisting of HN001 and *Bifidobacterium animalis* ssp. *lactis* 420 from early in pregnancy until 6 months postpartum, did not report effects on postpartum depressive and anxiety symptoms.^15^ This discrepancy may be due to the latter study using two probiotics, a different dose of HN001, the very low baseline levels of mental health symptoms in the study sample, and/or involving participants who were all overweight.^15^ These studies, nonetheless, collectively suggest that HN001 may be promising in supporting maternal mental health in females across pregnancy and the postpartum period, but more research is clearly needed.

Whether the effects of HN001 on the gut‐microbiota–brain axis and affective state are specific to reproducing females also remains to be determined. In male rats orally administered HN001, stress‐induced depressive‐like behaviors are reduced and basal levels of key brain neurotransmitters including serotonin, dopamine and norepinephrine are altered.^19^ In male Wistar–Kyoto rats, which are considered “stress‐sensitive” rats, orally administered HN001 did not affect anxiety‐like behaviors but did alter expression of the glutamate receptor (Grm4) in the amygdala.^20^ To our knowledge, no research exists on how HN001 may affect the gut‐microbiota–brain axis of nulliparous female rodents.

In addition, the brain changes significantly during pregnancy and the postpartum period, indicating that reproductive state is a critical factor to consider when looking at the gut‐microbiota–brain axis.^21^, ^22^, ^23^, ^24^, ^25^, ^26^ This reproduction‐related neuro‐ and glial‐plasticity is part of the normative physiological modifications occurring during the transition to motherhood (i.e., matrescence) and impacts many brain areas.^23^ Indeed, brain plasticity during pregnancy and the postpartum period produces a unique cellular environment that must be considered when developing interventions or treatments that support maternal well‐being, including those focused on maternal mental health.^27^, ^28^

Therefore, the primary aim of the current study was to determine whether the effects of chronic probiotic treatment with HN001 on the adult female rat gut‐microbiota–brain axis depend on reproductive state. To accomplish this, we used a laboratory rodent model involving a 2 × 2 experimental design (treatment × reproductive state) resulting in four groups of age‐matched female rats: (1) Virgin Control (VirginCON), (2) Virgin Probiotic (VirginPRO), (3) Maternal Control (MaternalCON), and (4) Maternal Probiotic (MaternalPRO). We focused on plasticity (neurogenesis, microglia, synaptophysin density) in the hippocampus and the cingulate gyrus of the cortex, two brain areas involved in affective state^29^ and well‐known to exhibit plasticity across pregnancy and motherhood.^23^, ^26^ We also investigated the effect of HN001 on maternal retrieval of offspring, based on our previous findings that pregnancy treatment with HN001 altered mother–infant interactions in rats.^10^ We hypothesized that chronic oral administration of HN001 from pregnancy through the early postpartum period would have different effects on the gut‐microbiota–brain axis of reproductively experienced females than on virgin females. We further hypothesized that in reproductively experienced females, HN001 treatment would modify maternal caregiving behaviors. We also expected associations between the abundance of gut microbes and neuroplasticity measures in the hippocampus and cingulate cortex of the females.^6^

## 2 MATERIALS AND METHODS

### 2.1 Animals

Thirty Long–Evans female rats (average body weight = 175 g, ~70 days old) and five adult male rats, used for breeding, were purchased from Janvier Labs (Le Genest‐Saint‐Isle, France). Animals were housed under standard laboratory conditions in a 12:12‐h light/dark schedule (lights on at 0700 h) with access to standard rat chow and tap water ad libitum. All experiments were approved by the Ethics Committee of the French Ministry of Research (authorization number: APAFiS #34181‐2021113014321334). The animal facility is licensed by the French Ministry of Agriculture (agreement D35‐238‐19). Upon arrival, animals were housed in same‐sex groups of two or three animals in transparent polyurethane cages for at least 1 week prior to the beginning of the experimental protocol. All efforts were made to minimize animal stress and pain.

**FIGURE 1:**
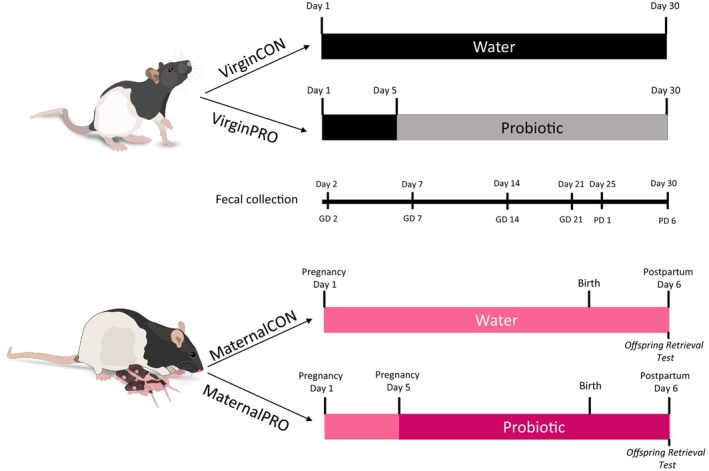
Experimental design. Age‐matched female rats were divided into four groups: (1) VirginCON—treated with water, (2)VirginPRO—treated with HN001, (3) MaternalCON—treated with water, and (4) MaternalPRO—treated with HN001. Day 1 of the study was the first day of the study in all females and corresponded with the first day of gestation in females in the MaternalCON and MaternalPRO. The probiotic treatment started on Day 5 of the study for VirginPRO and MaternalPRO females. Feces were collected at 6 time points throughout the study for microbiota analysis. This was done once prior to probiotic treatment on Day 2 and then on Days 7, 14, 25, and 30, which correspond to gestation days (GD) 2, 7, and 14, and postpartum days (PD) 1 and 6. On the final day of the study, females in the MaternalCON and MaternalPRO groups were tested for offspring retrieval, and the Virgin females were treated in the same way, but without offspring exposure. All females were euthanized after testing, and brains, caecum, and feces from the intestine were collected postmortem. (*n* = 5–6 per group; rat images freely available at github.com).

Prior to breeding and treatment, females were randomly divided into virgin and reproductively experienced groups, resulting in the following four groups: (1) VirginCON—virgin females given untreated drinking water (*n* = 6); (2) VirginPRO—virgin females given HN001 in their drinking water (*n* = 6); (3) MaternalCON—mated females given untreated drinking water (*n* = 9); (4) MaternalPRO—mated females given HN001 in their drinking water from pregnancy through the early postpartum period (*n* = 9). For a timeline of the experimental design, see Figure 1.

For breeding, two females and one male were paired. Vaginal smears were taken daily to determine if copulation had taken place as evidenced by the presence of spermatozoa (Gestation Day 1; GD1). After successful mating, the female was removed from the cage and singly housed. At the same time, a female in one of the Virgin groups was also singly housed. Seven females in the reproductively experienced groups did not successfully reproduce, so were removed from the study, leaving *n*s = 5–6 in the postpartum groups. The weights of the females were recorded weekly. Weight, size, and sex ratio of each litter was recorded within 24 h after parturition and at the end of the study. The birth weight was not available for one dam as she birthed on a day when technicians did not enter the lab. Within 24 h of parturition, the litters were each culled to eight offspring, containing four male, and four female offspring when possible.

### 2.2 Feces collection

Feces were collected prior to treatment and continued until the end of the experiment on Days 2, 7, 14, 21, 25, and 30 of the study. For collection of feces on Days 2–25, females were transferred to new cages with clean bedding, and the following Day 2 fecal pellets were collected with sterilized tweezers as previously described.^6^, ^30^ Upon collection, feces were stored in sterile 1.5‐mL tubes and frozen at −80°C until microbe analysis. At the time of terminal anesthesia at the end of the study (Day 30), feces were collected directly from the colon.

### 2.3 *L. rhamnosus* HN001 (HN001) administration

Preliminary assessment of bacteria viability of the HN001 samples was performed prior to treating the rats. To do this, lyophilized *L. rhamnosus* HN001 (gifted from Fonterra Cooperative Ltd, New Zealand), stored at −80°C, was resuspended in sterile 0.1% peptone solution or directly in tap water before serial dilution and plating in Agar MRS (Sigma) petri dish at 37°C to evaluate the number of colony‐forming unit (CFU) per mg of freeze‐dried powder. MRS agar, equilibrated at 45°C after sterilization, was quickly poured on 1 mL of diluted bacteria to include the bacteria into the medium and reduce exposure to oxygen. We quantified CFU/mg lyophilized bacteria from 1/100 million dilution performed in duplicate.

When suspended and diluted in 0.1% peptone and cultured for 48 h, the resuspended HN001 provided 6.8 × 10^8^ CFU/mg. However, to closely model oral exposure in rats, we suspended bacteria directly in water (no peptone) and immediately cultured in MRS Agar as above. Quantification revealed a reduced viability to 1.93 × 10^8^ CFU/mg. When suspended in water for 48 h before culture in a water bottle used for rats, a further reduction of viability was observed at 2.3 × 10^8^CFU/mg. We then chose to use 1 mg/100 mL of water for the experimental in vivo exposure.

The rats had unlimited access to their water bottle, containing untreated water (CON groups) or containing HN001 (PRO groups). Bottle contents were changed every 48–60 h and were weighed before and after changing to define the amount of fluid ingested and thus exposure to the probiotic, as well as to determine potential changes in water intake related to treatment or reproductive state. Fluid intake was calculated at four time periods of the study timeline: Week 1 (Study Days 5–8), Week 2 (Study Days 9–15), Week 3 (Study Days 16–21), and Week 4 (Study Days 22–29) (see Figure 1 for a timeline).

### 2.4 Offspring retrieval test

To investigate maternal response to offspring, the postpartum groups were tested for offspring retrieval, which was performed between 09:00 and 11:30 on Day 6 postpartum (the final day of the study) and 90 min prior to perfusion, as previously described.^6^ In brief, offspring and mothers were removed from the home cage for 10 min. Offspring were placed back in the home cage in the opposite corner to the original nest site. The mother was returned to the home cage containing her offspring, and maternal behaviors were video recorded for 15 min. Females in the Virgin groups were also removed from their cages for 10 min and replaced in their cages in the same fashion as females in the postpartum groups to control for handling before sacrifice. The durations and frequencies of the following maternal behaviors were scored from the video recordings by an experimenter blind to conditions using Stopwatch+ (developed by Kim Wallen, Emory University, USA): carrying offspring (picking up a pup at any time), sniffing offspring, licking offspring, quiescently nursing in any posture, nest building, and self‐grooming. The latency to the first pup retrieval and the time taken to retrieve all offspring to the original nest site from the start of the test were also recorded.

### 2.5 Euthanasia

Rats were deeply anesthetized with sodium pentobarbital (100 mg/kg IP) 90 min following the start of the offspring retrieval test or similar handling in virgin females. The rats were then rapidly perfused intracardially with 0.9% saline solution, the caecum and feces in the colon were collected and stored on dry ice, and females were then perfused with Somogyi solution (4% paraformaldehyde, 0.1% glutaraldehyde, and 15% saturated solution of picric acid). Brains were post‐fixed in 4% paraformaldehyde for 24 h, then cryoprotected up to 1 week in a phosphate–saline solution containing 30% sucrose, frozen on dry ice, and stored at −80°C. Brains were sectioned into 40‐μm sections in 10 series using a cryostat (Leica). Tissue was stored in cryoprotectant antifreeze solution (1% polyvinylpyrolidone, 30% sucrose, 30% ethylene glycol in phosphate‐buffered saline (PBS) 0.05 M) and maintained at −15°C until further processing.

### 2.6 Fecal HN001, *Bacteroidota*, *Bacillota*, and *Actinomycetota*

Fecal samples were used to investigate the abundance of HN001, *Bacteroidota*, *Bacillota*, and *Actinomycetota* (formerly known as *Bacteroidetes*, *Firmicutes*, and *Actinobacteria*, respectively). *Bacteroidota*, *Bacillota*, and *Actinomycetota* are three of the four major phyla of the gut microbiota.^31^ Caecum samples obtained at perfusion were also analyzed for the same three phyla and strain (HN001). See Supporting Information for details.

For DNA extraction from the feces and caecum, Macherey‐Nagel bacterial DNA extraction kits were used (60–80 mg per sample) as previously described.^12^ The extracted DNA was quantified with a NanodropTM 8000 spectrophotometer (Thermo Fisher). Each sample was then diluted to 1 ng/μL. Primers for the bacterial families were used based on previous work^26^ (Table S1). The abundance of HN001, *Bacteroidota, Actinomycetota, Bacillota*, and the total bacteria 16S RNA gene for normalization were investigated by quantitative polymerase change reaction (PCR). A specificity check was performed, replacing the sample with sterilized water. Cycle thresholds (Ct) were used to analyze the proportions of the HN001 strain and the three different phyla in the microbiota sample, and the results are presented as 2*e*(−∆∆Ct). One outlier (VirginPRO group) was removed from the HN001 group on Day 30 for being more than 3 SD above the group mean.

### 2.7 Brain immunohistochemistry

To investigate the effects of reproductive state and HN001 treatment on measures of neuroplasticity and microglia, synaptophysin and microglia densities were investigated in the cingulate gyrus and the dorsal and ventral hippocampus. Neurogenesis in the dentate gyrus (DG) of the hippocampus was also investigated with the endogenous marker for immature neurons (doublecortin; DCX). C‐fos expression was also quantified to determine the activation of cells after offspring interaction (Maternal groups) or not (Virgin groups) in the cingulate gyrus; the CA1, CA3, and DG of the hippocampus; and the medial preoptic area (MPOA—a site involved in active maternal caregiving behaviors^32^).

**FIGURE 2:**
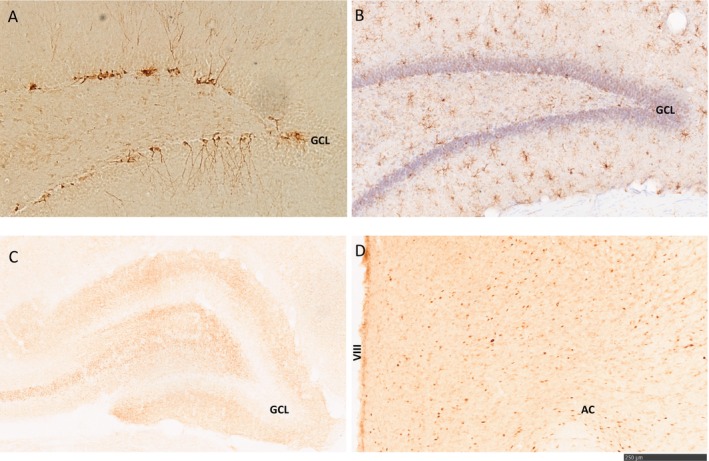
Representative photomicrographs of (A) DCX‐immunoreactive (DCX‐ir) cells, (B) Iba1‐ir cells, and (C) synaptophysin‐ir density in the dentate gyrus of the dorsal hippocampus taken at 10× objective, and (D) C‐Fos‐ir cells in the MPOA. Taken with a 10× objective. Scale bar = 250 μm. AC, anterior commissure; GCL, granule cell layer; MPOA, medial preoptic area; VIII, third ventricle.

For immunohistochemistry, tissue sections (one series of brain sections for each primary antiserum) were rinsed between steps in PBS and PBS plus 0.01% Triton X‐100. Tissue was incubated for 30 min at room temperature in 0.6% H_2_O_2_ followed by blocking for 30 min at room temperature in 5% normal donkey or goat serum. Tissue was incubated overnight at 4°C in rabbit anti‐DCX 1:5000 (Abcam, ab18723), mouse anti‐synaptophysin 1:500 (Sigma‐Aldrich, 5768), rabbit anti‐Iba1 1:5000 (Wako #19‐19741), or mouse anti‐Fos 1:500 (Abcam, ab208942). Sections were incubated in an appropriate secondary antiserum (1:500 biotinylated donkey anti‐rabbit or 1:500 biotinylated goat‐anti‐mouse) for 2 h at room temperature followed by processing using the avidin–biotin–peroxidase complex (Vectastain® Elite® ABC HRP Kit #PK‐6100; 1:1000; Vector Laboratories) and DAB (DAB kit; Vector Laboratories). Sections were mounted on Superfrost Plus slides (Fischer Scientific, Pittsburgh, PA), dried, dehydrated, and coverslipped with Permount (Fischer Scientific, Pittsburgh, PA). See Figure 2 for representative photomicrographs.

For quantification of hippocampal DCX‐immunoreactive (‐ir) cells, the number of DCX‐ir cells was counted under a 40× objective using an Olympus BX60 microscope by an experimenter naive to subject treatment groups, as previously described.^33^, ^34^ Cells were counted in the granule cell layer and subgranular zone (GCL/SGZ) of the hippocampus in both the dorsal (up to −4.92 bregma) and ventral regions. Cells were considered DCX‐ir if cell bodies were clearly labeled and relatively circular. DCX‐ir cells were further quantified and characterized based on their morphology in both the dorsal and ventral areas (75 cells per each region); DCX‐ir cells were categorized as either proliferative (no process or short process), intermediate (medium process with no branching) or postmitotic.^27^

For analysis of synaptophysin‐ir and Iba1‐ir cells in the cingulate gyrus (2.28–1.3 bregma) and the hippocampus (dorsal only, up to −4.92 bregma), photomicrographs were taken with an Olympus AX70 microscope and Cell^F software (Olympus) under 40× objective for synaptophysin and 10× objective for Iba1 by an experimenter naive to subjects' treatment groups. Two photomicrographs were taken of each of three slices of the cingulate gyrus, and each of two slices of the dorsal hippocampus (GCL/SGZ of the DG, CA1, and CA3) for synaptophysin and two slices of the dorsal and ventral CA3 for Iba1. (Note that the Iba1‐ir cells are uniformly distributed in the hippocampus.^35^)

Optical densities (OD) were obtained with ImageJ (NIH) software. For synaptophysin, the relative OD was calculated as the difference between optical density (grayscale after calibration) of the area of interest and the background of an adjacent region as previously described.^12^ For Iba1, photomicrographs were analyzed using ImageJ in order to determine the density (cingulate gyrus and CA3 regions) and the number of cells per area using the ImageJ cell counter plugin (https://www.unige.ch/medecine/bioimaging/information/tutorials-1/image-j-fiji). Cells were quantified if they were densely immunoreactive and relatively circular. Microglia morphology was quantified in the cingulate gyrus and GCL/SGZ based on.^28^ The four morphological states identified and quantified are as follows: (a) microglia with thin, ramified processes (thin); (b) microglia with thick, ramified processes (thick); (c) microglia with enlarged cell bodies and few short branches (stout/transitioning); or (d) microglia with enlarged cell bodies and no processes (ameboid). Sixty cells in both the dorsal DG and the cingulate gyrus (from 3 sections) and 40 cells in the ventral DG (from 2 sections) were quantified. Data for Iba1‐ir cell types is expressed as the percentage of total cells examined.

C‐Fos‐ir cells were counted in the cingulate gyrus, hippocampus (CA1, CA3, DG) and MPOA using QuPath (Open Software for Bioimage Analysis, https://qupath.github.io/). In brief, slides were scanned at 40× objective using HAMAMATSU scanner from H2P2 service platform (BIOSIT). Rectangular areas were selected within each brain region of interest: in the cingulate cortex three 300 × 700 pixels rectangle were applied in the right and left cingulate gyrus in three sections per animal; in the hippocampus two 300 × 500 pixels rectangles were applied in right and left CA1, CA3, DG in at least two sections per animal in the dorsal (total eight areas measured in each area of interest) and two sections in the ventral hippocampus (total six areas measured); in the MPOA, two 300 × 500 pixels regions were selected in each of the ventral lateral, ventral medial, dorsal medial and dorsal lateral areas of the MPOA in one section per animal (MPOA results in supplemental data due to *n* = 4 in one group). Cell nuclei were defined using QuPath extension and script developed by FAIIA platform (Thierry Pécot) (https://github.com/qupath/qupath-extension-stardist/releases/tag/v0.4.0), with pixel size set between 0.5 and threshold at 0.2. Using 13 selected representative areas, we used the “train object classifier” to confirm the detection of true nuclei and remove artifacts. Cells were defined if they were uniform in size, circular, and densely immunoreactive. Once the training was done, cell counting was run for each area and the mean of the number of cells was calculated for each brain area to obtain one measure per brain region per animal.

### 2.8 Statistical analysis

Statistical analysis was performed using the software Statistica 13 (Tibco Software). For body weight, fluid intake, and abundance of gut microbiota across timepoints, we used repeated measures factorial analysis of variance (ANOVA) tests with reproductive state (virgin vs. mother) and treatment (control vs. probiotic) as between subjects' factors. Because of the differences in fecal collection methods at the time of perfusion compared to at other times in the study, a factorial ANOVA was carried out on Day 30 gut microbiota abundance, and this day was not included in the repeated measures ANOVA of fecal microbes (for repeated measures ANOVA results including all fecal collection time points see Figure S3).

Measures of neuroplasticity were analyzed using 2 × 2 factorial ANOVAs with reproductive state (virgin vs. mother) and treatment (control vs. probiotic) as factors.

In the maternal females (MaternalCON and MaternalPRO groups), the analyses of litter weight at birth, sex ratio, culled litter weight at perfusion, and maternal behaviors in the offspring retrieval test were done with a one‐way ANOVA using treatment (control vs. probiotic) as the between‐subject factor.

Pearson correlations were conducted across all groups between the fecal microbiota and brain measures. Within and between group correlations were not considered because of the low sample size. Because of the relationship between synaptic plasticity and microglia in the hippocampus,^29^ correlations were performed between these brain measures in the dorsal hippocampus. In the maternal females (MaternalCON and MaternalPRO groups), Pearson correlations were also used to examine the relations between fecal microbiota abundance on the last day of the study, mothers' pup retrieval measures, and the brain measures across groups. Tukey's post hoc tests were only used after significant omnibus ANOVAs. Significance was set at *p* < 0.05. The data that support the findings of this study are available on request from the corresponding author, but are not publicly available due to privacy and ethical restrictions.

## 3 RESULTS

### 3.1 Body weight increased with pregnancy

At the start of the study, there were no significant differences in body weight among the four groups of females (*p* > 0.4). As expected, across pregnancy, females in the maternal groups weighed more than females in the virgin groups (main effect of reproductive state; *F*[1,20] = 191.01, *p* = 0.000001, *ηp* ^2^ = 0.91). On the day of brain extraction, age‐matched virgins weighed significantly less than dams (*F*[1,19] = 50.818, *p* = 0.00001, *ηp* ^2^ = 0.73; Figure S1). There were no other significant main effects or interaction effects on subjects' body or litter weights (*p*s >0.15).

### 3.2 HN001 dams birthed more offspring

MaternalPRO dams birthed significantly more offspring (*F*[1,8] = 5.480, *p* = 0.047, *ηp* ^2^ = 0.41; MaternalPRO mean litter size 13.4 ± 0.5, MaternalCON mean litter size 9.4 ± 1.6) but there were no significant differences in total litter weight (*F*[1,8] = 2.76, *p* = 0.14). There was also no difference between groups in the sex ratio of the litters at birth (*F*[1,8] = 2.25, *p* = 0.17). Culled litters at the end of the study did not differ in weight (*F*[1,9] = 0.85, *p* = 0.38).

### 3.3 Fluid intake is increased in pregnant females

As expected, dams consumed more liquid than virgin females from Day 15 until the end of the study (*p* < 0.02; significant day by reproductive state interaction: *F*[3,54] = 7.965, *p* = 0.0002, *ηp* ^2^ = 0.31; Figure S2). There was also a significant main effect of treatment (*F*[1,18] = 38.253, *p* = 0.00001, *ηp* ^2^ = 0.68), reproductive state (*F*[1,18] = 33.661, *p* = 0.00002, *ηp* ^2^ = 0.65), and day (*F*[3,54] = 72.411, *p* = 0.00001, *ηp* ^2^ = 0.80) on fluid intake. This indicates that females in the probiotic and/or maternal groups drank more fluid overall and that when collapsed across groups fluid intake increased from the beginning to the end of the study (see Figure S2 for details).

### 3.4 HN001 increases carrying of offspring

**FIGURE 3:**
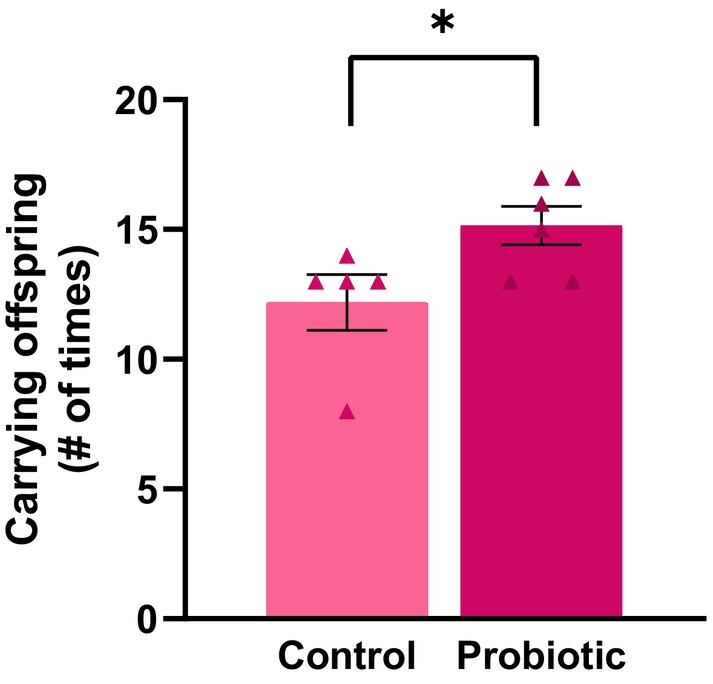
Mean (±SEM) number of times a dam carried her offspring during the offspring retrieval test. MaternalPRO females picked up and retrieved offspring more often than MaternalCON females (*p* = 0.04; *n* = 5–6 per group). *p < 0.05.

MaternalPRO females carried offspring more frequently compared to MaternalCON females (main effect of probiotic treatment *F*[1,9] = 5.450, *p* = 0.04, *ηp* ^2^ = 0.38; Figure 3). There were no other significant effects of probiotic treatment on maternal caregiving behaviors during the offspring retrieval test (*p*s >0.06; Table 1).

**TABLE 1:** Mean (±SEM) duration (seconds) of maternal behaviors scored from video recordings of the offspring retrieval test.

	MaternalCON	MaternalPRO
Sniffing offspring	18.56 ± 6.63	16.13 ± 4.74
Licking offspring	22.72 ± 6.25	22.58 ± 4.71
Nestbuilding	11.16 ± 4.20	29.71 ± 8.23
Self‐grooming	36.34 ± 9.07	21.48 ± 4.17
Carrying offspring	48.08 ± 11.87	62.50 ± 9.63

*Note*: There were no significant differences between groups, all *p*s > 0.05. None of the dams spent time quiescently nursing offspring during the 15‐min test (*n* = 5–6 per group).

### 3.5 HN001 treatment decreases abundance of *Bacteroidetes* at the end of the study

**FIGURE 4:**
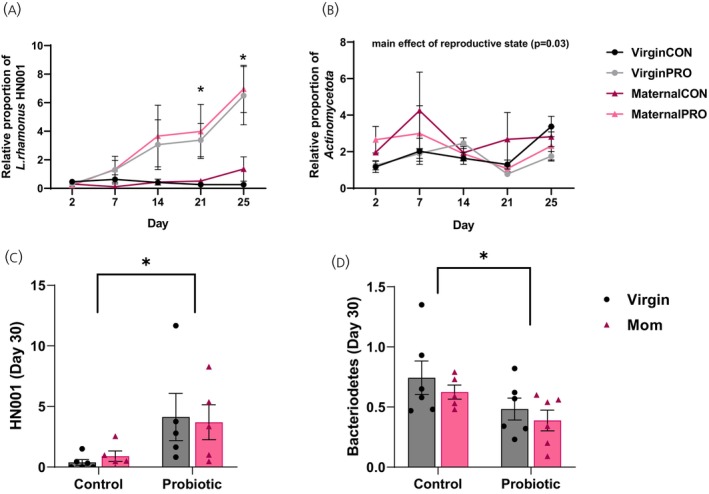
Mean (±SEM) ratio to the total abundance of bacteria in feces 2^−∆∆Ct^. (A) HN001 abundance was significantly greater in the gut of HN001‐treated females compared to untreated females on Day 21 (*p* = 0.05) and Day 25 (*p* = 0.0002) of the study. (B) From Day 2 to Day 25 of the study there was a significant main effect of reproductive state in *Actinomycetota* abundance (*p* = 0.03) with virgin females having less *Actinomycetota* in the gut than maternal females. (C) An analysis of microbiota composition from fecal samples obtained from the intestinal tract at the time of perfusion show that HN001 treatment resulted in a significant increase in the abundance of HN001 in the gut on Day 30 (*p* = 0.007) and (D) a significant decrease in the abundance of *Bacteroidetes* (*p* = 0.025) compared to untreated females, regardless of reproductive experience (*n* = 5–6 per group). *p < 0.05.

A repeated measures analysis of microbiota abundance from fecal samples obtained from the home cage of the female shows that HN001 abundance was significantly greater in the gut of HN001‐treated females compared to untreated females specifically on Day 21 (*p* = 0.05) and on Day 25 (*p* = 0.0002) of the study (interaction effect: *F*[4,76] = 5.5, *p* = 0.0006, *ηp* ^2^ = 0.22; Figure 4A). There was also a significant main effect of treatment (F[1,19] = 30.6, *p* = 0.00002) and day (*F*[4,76] = 7.00, *p* = 0.00007) on HN001 abundance in the gut. There was a significant main effect of reproductive state in *Actinomycetota* abundance across the study (Days 2–25) (*F*[1,20] = 5.43, *p* = 0.03, *ηp* ^2^ = 0.21; Figure 4B) with virgin females having less *Actinomycetota* in the gut than maternal females. There were no other significant main or interaction effects on the abundance of *Bacteroidota* (*p*s > 0.06), *Bacillota* (*p*s > 0.07), or *Actinomycetota* (*p*s > 0.07).

An analysis of microbiota abundance from fecal samples obtained from the intestinal tract at the time of perfusion shows that HN001 treatment resulted in a significant increase in the abundance of HN001 in the gut (*F*[1,18] = 9.17, *p* = 0.007, *ηp* ^2^ = 0.34; Figure 4C) and a significant decrease in the abundance of *Bacteroidetes* (main effect of treatment: *F*[1,19] = 5.6, *p* = 0.025, *ηp* ^2^ = 0.23; Figure 4D) in all probiotic‐treated females compared to control females, regardless of reproductive experience. There were no other main effects or interactions (*p* > 0.07).

### 3.6 Probiotic treatment modified the effect of reproduction on hippocampal neurogenesis and decreased synaptophysin density in the CA1

**FIGURE 5:**
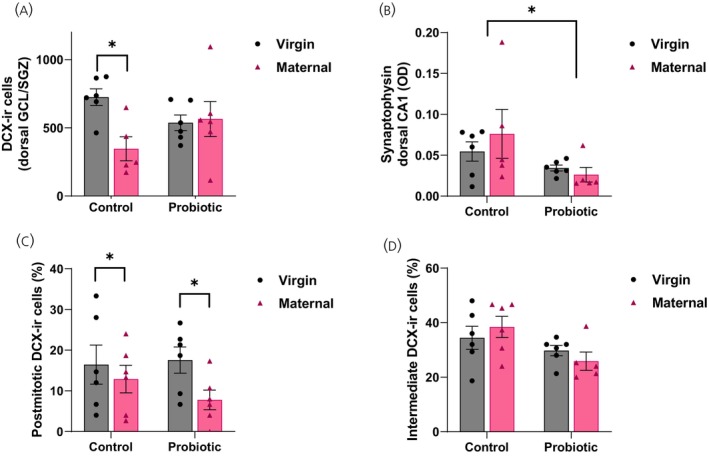
Mean (±SEM) (A) number of DCX‐immunoreactive (DCX‐ir) cells in the dorsal dentate‐gyrus, (B) optical density (OD) of synaptophysin‐ir in the dorsal CA1, (C) percentage of intermediate DCX‐ir cells, and (D) percentage of postmitotic DCX‐ir cells in the dorsal dentate gyrus. (A) MaternalCON females had significantly fewer immature neurons than VirginCON females (*p* = 0.04). (B) Probiotic‐treated females had significantly lower levels of synaptophysin density in the dorsal CA1 (*p* = 0.04). (C) There was a significant interaction effect for the number of intermediate immature neurons in the dentate gyrus (*p* = 0.04). (D) VirginCON and VirginPRO females had more postmitotic immature neurons than MaternalCON and MaternalPRO females (significant interaction, *p* = 0.02; *n* = 5–6 per group). *p < 0.05.

In the dorsal GCL/SGZ of the DG, MaternalCON females had significantly fewer DCX‐ir cells (i.e., immature neurons) than the VirginCON females (*p* = 0.04; significant reproductive state by treatment interaction: *F*[1,19] = 5.221, *p* = 0.03, *ηp* ^2^ = 0.22; Figure 5A). In the dorsal CA1, probiotic‐treated females had significantly lower density of synaptophysin‐ir (main effect of treatment *F*[1,18] = 5.019, *p* = 0.04, *ηp* ^2^ = 0.22; Figure 5B).

When looking at the type of dcx‐ir cells, there was a significant main effect of reproductive state on the number of postmitotic immature neurons in the dorsal GCL/SGZ (*F*[1,19] = 4.779, *p* = 0.04, *ηp* ^2^ = 0.20; Figure 5C), with virgin females having more of them than maternal females. There was also a significant interaction between reproductive state and probiotic treatment for the number of intermediate DCX‐ir cells in the dorsal GCL/SGZ (*F*[1,19] = 6.117, *p* = 0.02, *ηp* ^2^ = 0.24; Figure 5D) but post hoc tests revealed no significant differences between any pairs of groups (*p*s >0.09). There were no other significant effects on DCX‐ir cells in the GCL/SGZ or synaptophysin optical density in the hippocampus or cingulate gyrus (*p*s >0.07; Table 1).

### 3.7 Probiotic treatment and reproductive state alter microglia morphology

**FIGURE 6:**
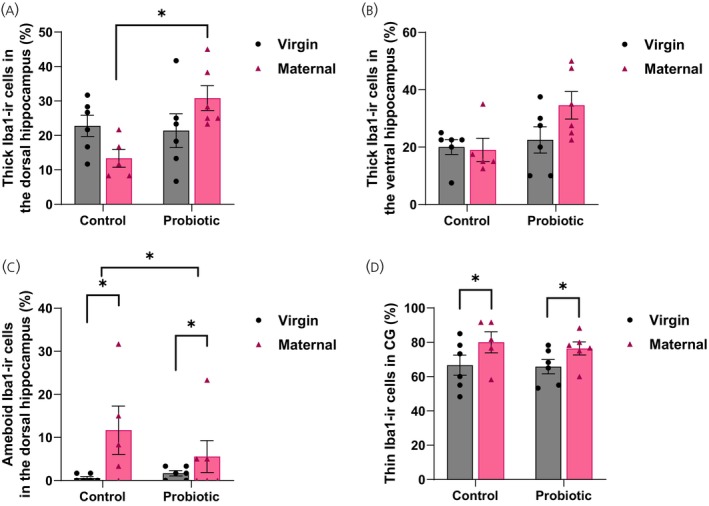
Mean (±SEM) percentage of (A) thick‐type Iba1‐ir cells in the dorsal hippocampus, (B) thick‐type iba1‐ir cells in the ventral hippocampus, (C) ameboid type Iba1‐ir cells in the dorsal hippocampus, and (d) thin‐type Iba1‐ir cells in the cingulate gyrus (CG). (A) In the dorsal hippocampus, MaternalPRO females had significantly more thick‐type Iba1‐ir cells than MaternalCON females (*p* = 0.02). (B) In the ventral hippocampus, VirginPRO and MaternalPRO females had more thick‐type Iba1‐ir cells than VirginCON and MaternalCON females (*p* = 0.04). (C) For the number of ameboid‐type Iba1 cells in the dorsal hippocampus, there was also a significant main effect of reproductive state (*p* = 0.03) and treatment (*p* = 0.04). (D) In the CG, VirginCON and VirginPRO females had significantly fewer thin‐type Iba1‐ir cells than MaternalCON and MaternalPRO females (*p* = 0.03; *n* = 5–6 per group). *p < 0.05.

In the dorsal hippocampus, MaternalPRO females had significantly more thick type Iba1‐ir cells than did MaternalCON females, but there was not a significant difference between VirginPRO and VirginCON females (*p* = 0.02; significant interaction effect: *F*[1,19] = 6.343, *p* = 0.02, *ηp* ^2^ = 0.25; Figure 6A). In the ventral hippocampus, probiotic‐treated females had more thick‐type Iba1‐ir cells than did the control females (main effect of treatment: *F*[1,19] = 4.797, *p* = 0.04, *ηp* ^2^ = 0.20; Figure 6B). There were also significant main effects of reproductive state (*F*[1,19] = 5.806, *p* = 0.03, *ηp* ^2^ = 0.23) and treatment (*F*[1,19] = 4.614, *p* = 0.04, *ηp* ^2^ = 0.20; Figure 6C) on the number of ameboid‐type Iba1 cells in the dorsal hippocampus. In the cingulate gyrus, virgin females, regardless of treatment, had significantly fewer thin Iba1‐ir cells than did the maternal females (main effect of reproductive state: *F*[1,19] = 5.654, *p* = 0.03, *ηp* ^2^ = 0.23; Figure 6D). There were no significant main or interaction effects of treatment or reproductive state on Iba1 cell types or Iba1‐ir density in the cingulate gyrus or hippocampus (*p*s > 0.06).

### 3.8 Reproductive state and C‐Fos expression in the hippocampus and CG

There were no significant main effects or interaction effects on C‐Fos expression in the CG, dorsal, or ventral hippocampus (*p*s > 0.1; Table 2).

**TABLE 2:** Mean (±SEM) for DCX‐ir cells, synaptophysin‐ir density, Iba1‐ir density, and C‐Fos‐ir cell number in the hippocampus and/or cingulate gyrus.

	VirginCON	VirginPRO	MaternalCON	MaternalPRO
*DCX‐ir cells*				
Ventral DG	677.00 ± 80.72	795.67 ± 74.08	788.00 ± 63.69	793.17 ± 112.75
*Synaptophysin‐ir density*				
CA3	0.106 ± 0.01	0.103 ± 0.02	0.153 ± 0.03	0.098 ± 0.02
Dorsal DG	0.120 ± 0.02	0.081 ± 0.01	0.122 ± 0.05	0.097 ± 0.01
CG	0.079 ± 0.02	0.067 ± 0.01	0.075 ± 0.01	0.070 ± 0.01
*Iba1‐ir density*				
Dorsal *hipp*	0.0005 ± 0.0001	0.0006 ± 0.0001	0.0005 ± 0.0001	0.0005 ± 0.00009
Ventral *hipp*	0.0002 ± 0.00008	0.0003 ± 0.00006	0.0003 ± 0.00006	0.0003 ± 0.00006
CG	0.0005 ± 0.00008	0.0005 ± 0.00006	0.0005 ± 0.00006	0.0006 ± 0.00006
*c‐Fos‐ir cells*				
Dorsal *hipp*	6.06 ± 1.28	5.35 ± 1.04	3.53 ± 0.88	6.92 ± 1.40
Ventral *hipp*	5.06 ± 1.06	4.81 ± 1.00	5.12 ± 1.17	5.49 ± 0.59
CG	31.88 ± 6.77	31.19 ± 9.92	26.80 ± 7.01	32.25 ± 7.95

*Note*: There were no significant main or interaction effects on these measures (*n* = 5–6 per group).

Abbreviations: CG, cingulate gyrus; DCX‐ir, DCX‐immunoreactive; DG, dentate gyrus; *hipp*, hippocampus; OD, optical density.

### 3.9 Correlations between gut microbiota, brain measures, and maternal behavior

**FIGURE 7:**
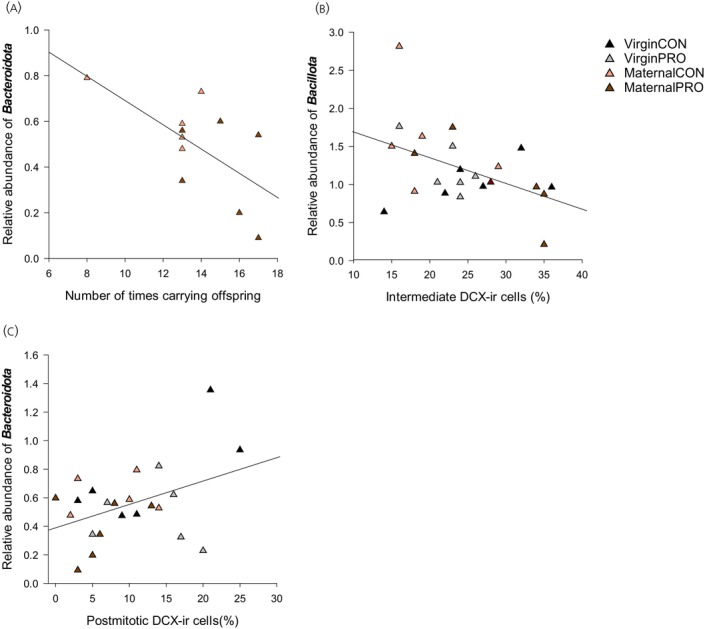
Correlations between gut microbiota, brain measures, and maternal behavior. (A) In MaternalCON and MaternalPRO females, there was a significant negative correlation between the number of times picking up or retrieving offspring and the relative abundance of *Bacteroidota* (*r* = −0.632, *p* = 0.037). (B) There was also a significant negative correlation between the relative abundance of *Bacillota* in feces on Day 30 and the number of intermediate DCX‐immunoreactive (DCX‐ir) cells in the dorsal granule cell layer and subgranular zone (GCL/SGZ) (*r* = 0.416, *p* = 0.048). (C) There was a significant positive correlation between the relative abundance of *Bacteroidota* on Day 30 and postmitotic DCX‐ir cells in the dorsal GCL/SGZ (*r* = −0.456, *p* = 0.029).

In maternal females, there was a significant negative correlation between the frequency dams spent carrying offspring and the relative abundance of fecal *Bacteroidota* on Day 30 (*r* = −0.632, *p* = 0.037; Figure 7A). In all females, there was a significant positive correlation between the relative abundance of *Bacteroidota* on Day 30 and postmitotic dcx‐ir cells in the dorsal GCL/SGZ (*r* = 0.416, *p* = 0.048; Figure 7B). There was also a significant negative correlation between the relative abundance of fecal *Bacillota* on Day 30 and the number of intermediate dcx‐ir cells in the dorsal GCL/SGZ (*r* = −0.456, *p* = 0.029; Figure 7C). There was a trend towards significant correlations between the relative abundance of *Bacillota* in feces on Day 30 and the percentage of thick‐type Iba‐ir cells (*r* = −0.41, *p* = 0.052) and thin‐type Iba‐ir cells in the ventral hippocampus (*r* = 0.41, *p* = 0.051; Figure S3).

**FIGURE 8:**
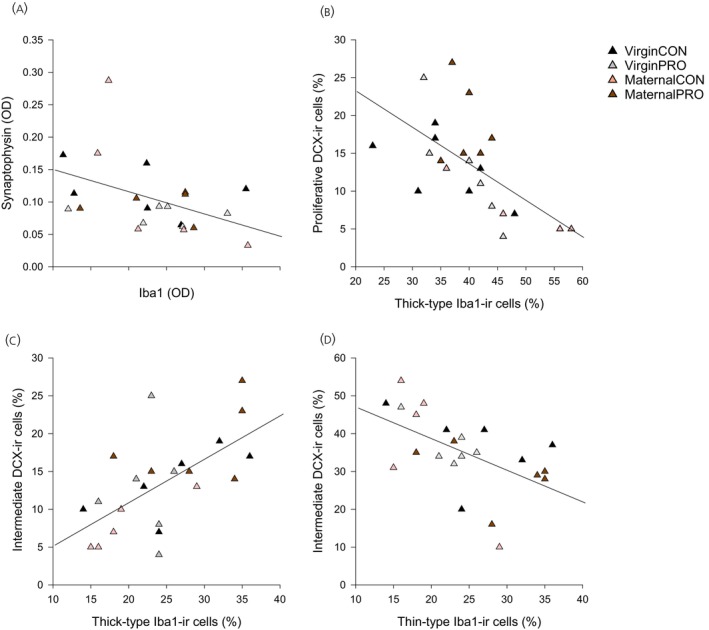
Correlations between brain measures. (A) There was a significant negative correlation between the density (OD) of Iba1‐ir cells in the hippocampus and synaptophysin‐ir in the dentate gyrus (DG, *r* = −0.442, *p* = 0.039). Correlations between thick‐type Iba1‐ir cells and (B) percentage of proliferative DCX‐immunoreactive (DCX‐ir) cells (*r* = −0.609, *p* = 0.002) and (C) intermediate DCX‐ir cells (*r* = 6203, *p* = 0.002). (D) Thick‐type Iba1‐ir cells were also negatively associated with intermediate DCX‐ir cells in the dorsal hippocampus (*r* = −0.539, *p* = 0.008; *n* = 5–6 per group).

In the dorsal hippocampus, there was a negative correlation between the density of Iba1‐ir cells and synaptophysin‐ir (*r* = −0.442, *p* = 0.039; Figure 8A) as well as correlations between thick‐type Iba1‐ir cells and the percentage of proliferative DCX‐ir cells (*r* = −0.609, *p* = 0.002; Figure 8B) and intermediate DCX‐ir cells (*r* = 6203, *p* = 0.002; Figure 8C). Thick‐type Iba1‐ir cells were also negatively associated with intermediate DCX‐ir cells in the dorsal hippocampus (*r* = −0.539, *p* = 0.008; Figure 8D). There were no other significant correlations between measures (*p*s >0.07).

## 4 DISCUSSION

There is a growing need to understand the gut‐microbiota–brain axis during pregnancy and the postpartum period, particularly in its role in maternal wellbeing. In this study, we targeted the gut microbiota with *L. rhamnosus* HN001, a probiotic shown to improve depressive‐ and anxiety symptoms in human mothers^18^ and which we previously showed can decrease anxiety‐related behaviors in laboratory rat dams.^10^ The current study aimed to determine whether the effects of HN001 on aspects of the gut microbiota and brain were specific to females with reproductive experience or whether the same effects were evident in all adult females, regardless of reproductive state.

Main findings of the current study show that HN001 administration lowers *Bacteroidota* abundance in the gut regardless of reproductive state. In HN001‐treated dams, there was an increase in the number of times offspring were carried, and this was negatively correlated with *Bacteroidota* abundance in the dam's gut. HN001‐treated dams also had more immature neurons in the hippocampus and significantly more thick‐type microglial cells in the dorsal hippocampus compared to control dams. HN001‐treated females, regardless of reproductive experience, also had lower density of synaptophysin immunoreactivity in the CA1 and more thick‐type microglia cells in the ventral hippocampus compared to control females.

### 4.1 HN001 modifies gut microbiota

The benefits of oral probiotics are predicated on their ability to alter microbes of the gut. We confirmed that providing female rats (virgin or pregnant) HN001 in their drinking water for 25 days increased the abundance of *L. rhamnosus* HN001 in their feces by the end of treatment. This is consistent with our previous study involving reproducing female rats given HN001 overnight for ~30 days starting at insemination through the first 10 days postpartum.^10^ Here we further found that HN001 treatment reduced the abundance of *Bacteriodota* by the end of the study in females. We did not find this same reduction in *Bacteriodota* in our previous work, but this is probably due to a number of differences between our studies. These differences include the duration of treatment (25 vs. 30 days); the number of fecal samples obtained, such that the present study's six samples increased the possibility to detect a “signal” compared to our previous study that only obtained two samples (one in pregnancy, one postpartum); and how long each day females had access to HN001 in their drinking water (continually vs. overnight). Apparent discrepancies may also be due to the different groups of animals used, with our previous study only including reproducing females, and the techniques for analysis of fecal microbiota with our previous study's full gut microbiome analysis (16sRNA), compared to the targeted microbes measured herein.^10^ The current study also confirmed that gut microbes differ between virgin and reproducing female rats,^11^, ^12^ such that virgin females had less gut *Actinomycetota* than did the maternal females. These differences are likely a result of the many hormonal and physiological fluctuations associated with reproduction.

We also found lower *Bacteriodota* on Day 30 in HN001‐treated females, regardless of reproductive state. *Bacteroidota* are diverse phyla of bacteria recognized for their antimicrobial and probiotic properties. Outside of the peripartum period, research shows that *Bacteroidota* play a vital role in the digestion of complex carbohydrates, leading to the production of short‐chain fatty acids, which are essential for maintaining the gut barrier and exerting anti‐inflammatory effects.^36^ They also cooperate with other gut microbes to support nutrient absorption and gut health. Although a decrease in these microbes in the current study may imply an increased risk of dysbiosis in gut homeostasis, it also may indicate reorganization of gut bacteria with the addition of HN001.

### 4.2 Reproductive experience modifies *Acinomycetota* abundance in the gut

We found that reproductive state affected *Actinomycetota* abundance in the gut with virgin females having less *Actinomycetota* than maternal females. *Actinomycetota* are vital for the development and maintenance of gut homeostasis, playing a role in gut permeability, immune system function, metabolism, and the gut–brain axis.^31^, ^37^ There is limited research on how healthy pregnancy and motherhood impact *Actinomycetota* or other gut bacteria. One recent study in humans reports that *Actinomycetota* abundance increases from the second to third trimesters of pregnancy^38^ but this research did not include nonpregnant controls. There are also reports that an increase in the abundance of *Actinobacteria* and *Bacteroidota* in early pregnancy may be related to a lower risk of gestational diabetes mellitus during the second trimester, suggesting an important role for these bacteria in maintaining a healthy pregnancy.^37^

### 4.3 HN001 and maternal offspring‐directed care

We found that HN001‐treated dams carried offspring more frequently than did control dams during the offspring retrieval test on Day 6 postpartum. Our prior study^10^ also found that pregnancy through early postpartum HN001 treatment affected maternal caregiving behaviors, in particular the treated dams showing less stability and more disrupted caregiving across test days. Because offspring development is optimized by sensitive and efficient caregiving,^39^ the higher‐frequency pup carrying made by HN001‐treated dams in the present study may reflect inefficiency and suboptimal care. On the other hand, one could instead consider this increased carrying of offspring by HN001‐treated mothers as somehow adaptive and reflect changes in their sensitivity and responsiveness to possible alterations to their HN001‐exposed pup cues, thus improving their dyadic interactions. It should also be noted that in the present study there were no other differences between groups in other aspects of maternal offspring‐directed care or the time it took dams to retrieve offspring to the nest during the retrieval test. More in‐depth investigation is needed to determine how HN001 may impact maternal offspring‐directed care in rats, but our two studies collectively suggest its effects may be subtle.

We found a negative correlation between the frequency of carrying offspring and the fecal abundance of *Bacteroidota* in maternal females. Our previous research, investigating stress and SSRI treatment in pregnancy, did not find this relationship,^6^ though this apparent discrepancy between these study findings may be due to the timing of the offspring retrieval test, postpartum Day 2 versus postpartum Day 6, or the strain of rat used in the studies. Regardless, there is a growing body of literature showing that the composition of the maternal gut microbiota can mediate aspects of offspring brain and behavior development, such as the development of social behavior.^40^, ^41^ Very few of these studies have considered the role that variation in the maternal gut microbiota may have on maternal offspring‐directed behavior^6^, ^10^ but recent research shows that there is microbiota control of maternal behavior, particularly after giving an *Escherichia coli* strain that is pathogenic to the dams.^42^ It remains to be determined how more normative changes in gut microbe diversity and content can modify maternal care of offspring.

Lastly, it is worth mentioning that HN001‐treated dams gave birth to more pups (although all litters were then culled to 8 pups) compared to dams not treated with HN001. There is no obvious explanation for these findings, which we did not find in our prior study giving HN001 in dams' drinking water overnight starting at conception,^10^ but perhaps pregnancy HN001 administration maintained the viability of more fetuses in the current study partly by improving various aspects of placental function.^43^

### 4.4 HN001 modifies hippocampal neurogenesis

In the current study, HN001 treatment from early pregnancy through to the early postpartum period prevented the decrease in immature neurons (i.e., decreased neurogenesis) in the DG compared to that observed in the non‐treated rat dams. The normative reduction in hippocampal neurogenesis in the rat dam during late pregnancy and the early postpartum week is well documented.^25^, ^26^, ^44^, ^45^, ^46^ Previous research, although limited, has pointed to a role of corticosterone in modifying rat hippocampal neurogenesis during the early postpartum period. For example, postpartum adrenalectomy and low‐dose corticosterone replacement can limit the decrease in hippocampal neurogenesis measured in the early postpartum period.^44^ In line with this, high nursing demand, instilled by providing rat dams with food‐deprived pups every 12 h, both increases levels of circulating corticosterone in dams and reduces cell proliferation in the DG.^47^ This suggests that HN001 may reduce corticosterone levels in the postpartum period and, thus, increase hippocampal neurogenesis in dams.

HN001 effects on neurogenesis in the hippocampus of the dam may also be related to alterations in other modulators of hippocampal neurogenesis during pregnancy and postpartum, such as estradiol, allopregnanolone, and serotonin.^10^, ^25^, ^48^, ^49^, ^50^, ^51^ We have yet to understand the mechanism by which HN001 actions on the gut microbiome may alter plasticity in the hippocampus and elsewhere in the brain. In addition, research is needed to confirm the role of these new hippocampal neurons in the mother, or how the rate of neurogenesis in the maternal hippocampus relates to any behavioral outcomes or measures of mental health in the dam.^18^, ^26^

At the same time point in pregnancy characterized by decreased neurogenesis in the DG, previous research reveals an increase in synaptophysin density.^25^ This demonstrates that there are multiple levels of neuroplasticity that need to be explored, as there may be compensatory effects for decreased hippocampal neurogenesis in the peripartum period to some yet, unknown, brain functions. Indeed, in the current study, we found that the ability of HN001 to prevent the reduction in hippocampal neurogenesis was accompanied by decreased synaptophysin density in the CA1 region of the hippocampus. This was true for both virgin and maternal rats. This finding shows, as noted above, that HN001 administration appears to alter various aspects of hippocampal plasticity.

### 4.5 HN001, reproductive experience, and microglia

Microglia have many roles within the hippocampus, from regulating neurogenesis and synaptogenesis to modulating the release of cytokines.^52^, ^53^ In the present study, HN001 administration resulted in significantly more thick‐type microglial cells in the dorsal hippocampus compared to non‐treated dams. These same effects were not seen between virgin treated and non‐treated groups. Both reproductive experience and probiotic treatment also increased ameboid‐type microglia in the dorsal hippocampus. In the ventral hippocampus, probiotic‐treated females, regardless of reproductive experience, had more thick‐type microglial cells than did the non‐treated females. This shows that reproductive experience and the subregion of the hippocampus are important when investigating the effects of HN001 on microglia morphology.

These reproductive state differences in how HN001 affects microglia in the hippocampus could be related to the hormone changes across female reproduction that also modulate the response of dams to their offspring. However, it has yet to be determined how the gut microbiota and the hormones of pregnancy, birth, and postpartum may converge upon microglia of the maternal hippocampus.^54^ Interactions among gut microbiota, circulating steroid hormones, and microglia activation exist in nulliparous female mice^55^ and may provide some insight. Adult female hippocampal microglia have the capacity to express estrogen receptor alpha and progestin receptors,^56^, ^57^ and estradiol and progesterone can affect hippocampal microglia activation.^58^, ^59^ In addition, probiotics treatment can influence circulating estradiol and progesterone levels even in ovariectomized females.^60^, ^61^, ^62^ Perhaps HN001 treatment altered circulating steroid hormone levels in all females in our study, but particularly in our already endocrinologically labile reproducing females, resulting in the more widespread changes in hippocampal microglia activation in this group.

Microglia with thickened branches or no branches (ameboid) are thought to be more activated and engaging in inflammatory signaling during early‐life development.^63^ The exact role of these microglia in the adult female hippocampus is less clear, with some reports suggesting that these cells are a nascent form of ramified microglia.^64^, ^65^ It also remains to be determined how changes in microglia morphology with HN001 may be affecting neuroplasticity. Given that Iba1 is a microglia macrophage‐specific calcium‐binding protein,^64^ it may be that these changes in microglia in the dorsal hippocampus of the maternal HN001‐treated females are linked to the changes in neurogenesis via the phagocytotic properties of these Iba1 microglia cells. In line with this, in the current study, there was a positive correlation between thick‐type microglia and intermediate immature neurons, and a negative correlation between thick‐type microglia and proliferative immature neurons in the dorsal hippocampus. However, these correlations were not specific to dams. There was also a negative correlation between the density of microglia and synaptophysin in the DG of female rats.

It is important to note that previous work showed a reduction in microglia cell density in the hippocampus and cingulate gyrus across pregnancy compared to virgin females.^35^ Our findings did not replicate these results, and in some cases, we observed the opposite effect. For example, in the cingulate gyrus, virgin females, regardless of probiotic treatment, had significantly fewer thin Iba1‐ir cells than maternal females. While there is a growing body of research on the effects of reproductive experience on microglia, differences in findings once again may be due to rat strain differences^66^ given that the previous work showing a reduction used Sprague–Dawley females^35^ while the current work used Long–Evans females.

## 5 CONCLUSIONS

There is growing interest in understanding how the gut microbiota may promote maternal health. In the current study, we targeted the gut microbiota with the probiotic HN001 from early pregnancy through the early postpartum period, and at matched time points in age‐matched virgin females in order to begin to understand how HN001 differentially affects the gut‐microbiota–brain axis across female reproductive states. Our results show that HN001 administration as well as reproductive state, alone and together, can influence aspects of the gut‐microbiota–brain axis.

The mechanisms underlying how HN001 affects the gut‐microbiota–brain axis of the mother, and adult female, in general, remain to be determined. It may be that HN001 actions are related to the potential of HN001, similar to other *L. rhamnosus* strains, to enhance beneficial bacteria in the gut, resulting in the production of short‐chain fatty acids which have neuroprotective and anti‐inflammatory properties, as well as HN001's yet to be determined potential to alter the HPA axis and tryptophan metabolism,^67^ both of which have been associated with hippocampal neuroplasticity and caregiving behaviors.^26^, ^48^

Although it is unclear how hormonal fluctuations across the peripartum period can impact the gut‐microbiota–brain axis and probiotic influences on it,^68^ the current research suggests that, indeed, physiological and microbial interactions are key factors that can impact plasticity in the maternal brain. Further research is needed to determine the relevance of these findings for maternal wellbeing in women, but our results point to a valuable role of the gut‐microbiota–brain axis during the transition to motherhood.

## AUTHOR CONTRIBUTIONS

**Jodi L. Pawluski:** Conceptualization; investigation; funding acquisition; writing – original draft; methodology; validation; writing – review and editing; formal analysis; project administration; supervision; data curation; software; visualization. **Khadidja Kacimi:** Investigation; data curation; methodology; validation. **Cai Zhang:** Investigation; data curation; methodology; validation. **Laetitia Guillot:** Supervision; data curation; methodology; writing – original draft; investigation; formal analysis. **Aliocha Lo Guidice:** Methodology; investigation; data curation. **Thierry D. Charlier:** Supervision; resources; project administration; writing – original draft; conceptualization; methodology; software; visualization. **Joseph S. Lonstein:** Conceptualization; investigation; funding acquisition; writing – original draft; writing – review and editing; methodology; resources; visualization.

## FUNDING INFORMATION

This project was funded in part by the Biostime Institute for Nutrition and Care‐Geneva Funding programs awarded to JLP and JSL.

## CONFLICT OF INTEREST STATEMENT

JLP has consulted for Biogen Therapeutics and is on the Advisory Board of Strategies for Moms. All other authors declare no conflicts of interest.

## Supporting information


**Data S1:** Supporting Information.

## Data Availability

The data that support the findings of this study are available on request from the corresponding author. The data are not publicly available due to privacy or ethical restrictions.
