# Supplementary material for: Reproductive experience influences the effects of Lactocaseibacillus rhamnosus HN001 on gut microbiota and hippocampal plasticity in female rats
Source: J Neuroendocrinol. 2025 Jul 22;37(12):e70068. doi: 10.1111/jne.70068 (PMC12678013; doi:10.1111/jne.70068)
Supplement: Supplementary file 1 — Data S1: Supporting Information. [file JNE-37-e70068-s001.docx]

**Supplemental Data**

**Results**

**Caecum microbiota.** A one-way ANOVA revealed a main effect of reproductive state on *L.rhamnosus* HN001 content in the caecum ( F(1, 17)=9.0016, p=.00805) with virgins having more *L.rhamnosus* HN001 than mother rats. There was a tendency for an effect of treatment on *Bacillota* abundance in the caecum (F(1, 19)=3.8737, p=.06382) with probiotic treated females having less firmicute abundance. There was no significant effect of treatment or reproductive state on abundance of *Bacteroidota* (0.158 < p >0.176) or *Actinomycetota* ( 0.121 < p > 0.440). There were no other significant main or interaction effects in abundance of microbiota tested in the caecum (n=4-6/group).

**Reproductive state and C-Fos expression in the MPOA.** There was a trend towards to significant increase in C-Fos expression in maternal animals compared to virgin females (F(1, 16)=4.2762, p=.05522), although note for the MPOA there was only an n=4-6 per group.

**Figure S1.** Mean (+/-SEM) body weight (g) during the study. At the start of the study there were no significant differences in body weight among groups (p’s > 0.4). As expected, across pregnancy, females in the maternal groups weighed more than females in the virgin groups (p=.000001). At the end of the study, virgin females weighed significantly less than maternal females (p=.00001).

**Figure S2.** Mean (+/-SEM) fluid intake during the study. Dams (MaternalCON and MaternalPRO) consumed more liquid than virgin females from Day 15 until the end of the study (p’s < 0.02; significant day by reproductive state interaction F(3, 54)=7.9650, p=.00017). There was also a significant main effect of treatment (F(1, 18)=38.253, p=.00001), reproductive state (F(1, 18)=33.661, p=.00002), and day (F(3, 54)=72.411, p=0.00001 ) on fluid intake. *denotes main effect of reproductive state.

It is interesting to note that fluid intake varied over the course of the study. This may be for a number of reasons such as slight changes in humidity, temperature, or activity in the colony room or animal facility. In addition, the decrease in fluid intake at day 21 of the study may be related to the effects of physiological changes of late pregnancy or changes in activity in preparation for parturition.

**Figure S3.** Correlations between Iba1-ir cell types and *Firmicutes* (*Bacillota*) in the gut at the end of the study (Day 30). A) There was a negative correlation between thick-type Iba1-ir cells in the ventral hippocampus and *Bacillota* abundance in the gut on Day 30 of the study (r= -0.41, p=0.05) and B) a positive correlation between thin-type Iba1-ir cells in the ventral hippocampus and *Bacillota* abundance in the gut on Day 30 of the study (r= 0.41, p=0.05).


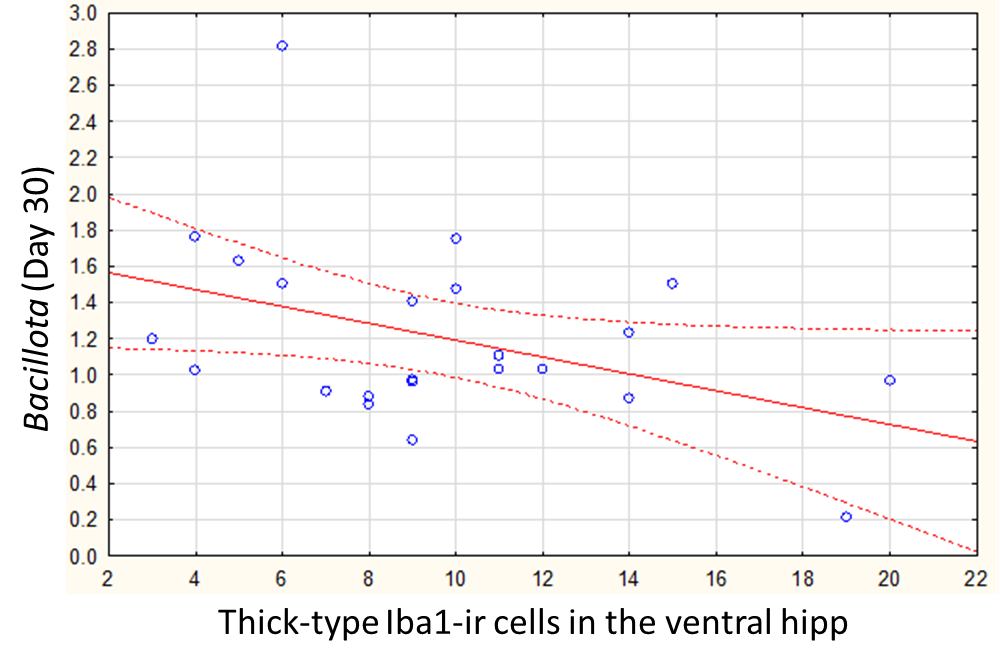
A)

**
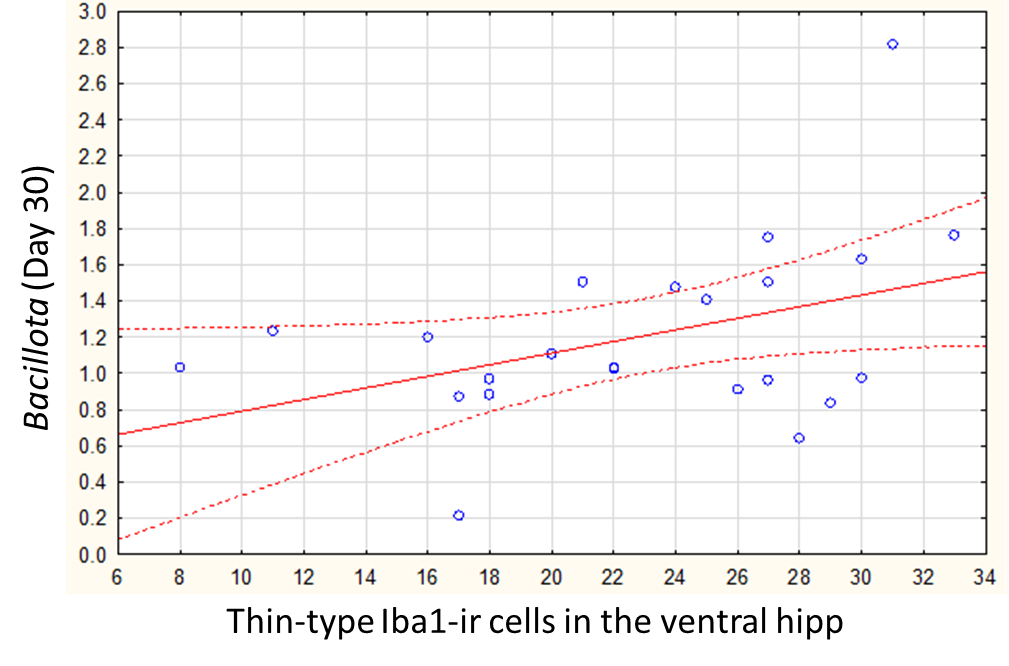
**B)

**Figure S4. Gut microbiota analysis of all time points in a repeated measures design.** Probiotic-treated females (MaternalPRO and VirginPRO) had a significantly greater abundance of faecal *L. rhamnosus* HN001 in faeces than did control females (MaternalCON and VirginCON ) (main effect of treatment: F(1, 18) = 33.541, p = 0.00002, ηp2 = 0.65 ; Figure S3A) and a lower abundance of *Bacteroidota* than control females (main effect of treatment: F(1, 20) = 4.594, p = 0.04, ηp2 = 0.19; Figure S3B). As expected, there was a significant time by treatment interaction (F(5, 90)=2.970, p=0.02, ηp2 = 0.14 ) with *L. rhamnosus* HN001 abundance significantly higher on days 25 and 30 of the study than at the beginning of the study (ps < 0.04). Collapsed across groups, there was a significant decrease in the abundance of *Bacteroidota* on Day 30 (PD6 for maternal females) compared to all other times (0.05 > ps > 0.004, main effect of time: F(5, 100) = 7.636, p = 0.000001, ηp2 = 0.28 ) and a decrease in the abundance of *Actinomycetota* on Day 30 (PD6 for maternal females) compared to Day 21 (GD21 for maternal females) (p = 0.036, main effect of time: F(5, 100) = 2.613, p = 0.03, ηp2 = 0.12 ; Figure S3C). There were no significant main or interaction effects on the abundance of faecal *Bacillota* and no other significant main or interaction effects (ps > 0.1; Figure S3D).

**Figure S5. Representative photomicrograph of an ameboid-type microglia cell (blue arrow) and a thick-type microglia cell (orange arrow) in the hippocampus. (20x objective)**

**
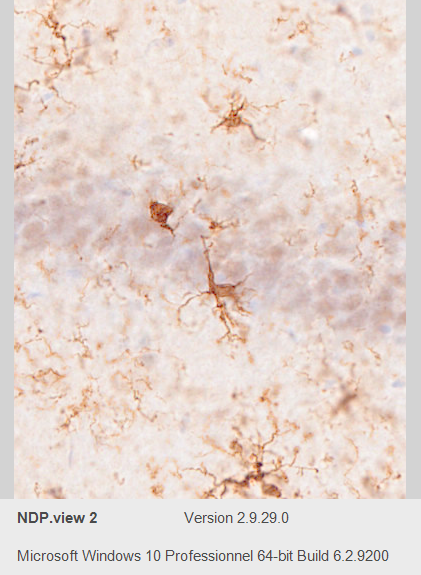
**

**Table S1. Primers used for PCR.**

The following primers were used:

Bacteroidota Bac960F (GTTTAATTCGATGATACGCGAG) 122 ; Bac1100R (TTAASCCGACACCTCA CGG)122.

Bacillota Firm934F (GGAGYATGTGGTTTAATTCGAAGCA) 126;Firm1060R (AGCTGACGACAACCATGCAC) 126.

Actinomycetota Act664F (TGTAGCGGTGGAATGCGC) 277 ; Act941R (AATTAAGCCACATGCTCCGCT) 277

Universal 926F (AAACTCAAAKGAATTGACGG) 136 ; 1062R (CTCACRRCACGAGCTGAC) 136 .

HN001For = 5’-cgct tAggActcAggAtAcA-3’

HN001rev = 5’-gcttgcgtcAgAttttcAgtA-3
